# Associations of impulsivity, hyperactivity, and inattention with nonsuicidal self-injury and suicidal behavior: longitudinal cohort study following children at risk for neurodevelopmental disorders into mid-adolescence

**DOI:** 10.1186/s12888-022-04311-5

**Published:** 2022-11-03

**Authors:** Olivia Ojala, Ralf Kuja-Halkola, Johan Bjureberg, Anna Ohlis, Martin Cederlöf, Eva Norén Selinus, Paul Lichtenstein, Henrik Larsson, Sebastian Lundström, Clara Hellner

**Affiliations:** 1 Centre for Psychiatry Research, Department of Clinical Neuroscience, Karolinska Institutet, & Stockholm Health Care Services, Norra Stationsgatan 69 7th fl, SE-11364 Stockholm, Sweden; 2 Department of Medical Epidemiology and Biostatistics, Karolinska Institutet, Stockholm, Sweden; 3 Department of Psychology, Stanford University, Stanford, CA USA; 4 Department of Global Public Health, Karolinska Institutet, Stockholm, Sweden; 5 Region Vastmanland – Uppsala University, Centre for Clinical Research, Vastmanland Hospital, Vasteras, Sweden; 6 The Swedish School of Sport and Health Sciences, Stockholm, Sweden; 7 School of Medical Sciences, Örebro University, Örebro, Sweden; 8 Gillberg Neuropsychiatry Centre, Institute of Neuroscience and Physiology, University of Gothenburg, Gothenburg, Sweden; 9 Centre for Ethics, Law and Mental health (CELAM), Institute of Neuroscience and Physiology, University of Gothenburg, Gothenburg, Sweden

**Keywords:** Self-injury, Suicidal behavior, Neurodevelopmental disorders, ADHD, Adolescence

## Abstract

**Background:**

The knowledge of how the separate Attention-Deficit/Hyperactivity Disorder (ADHD) subdimensions (impulsivity, hyperactivity, and inattention) are associated with nonsuicidal self-injury (NSSI) and suicidal behavior (SB) is limited. The objective of this study was to investigate the associations of childhood ADHD subdimensions with NSSI and SB in children at risk of neurodevelopmental disorders (NDDs; including ADHD).

**Methods:**

The sample (N = 391) included twin pairs where at least one twin screened positive for at least one NDD or common comorbidity at age 9 or 12. Data on ADHD subdimensions was collected through a telephone interview with a caregiver/legal guardian at age 9 or 12, and data on NSSI and SB was collected through an in-person clinical assessment at age 15. The associations between the ADHD subdimensions and NSSI or SB were tested in three different models: (1) univariable, (2) together with the other ADHD subdimensions, and (3) in a confounder-adjusted model including other NDD symptoms in addition to ADHD subdimensions, for NSSI and SB separately.

**Results:**

A total of 32 (8.2%) adolescents reported life-time engagement of NSSI, and 18 (4.6%) SB. Childhood impulsivity was associated with SB and childhood inattention with NSSI, in all models. Hyperactivity was not meaningfully associated with any of the outcomes.

**Conclusion:**

Impulsivity and inattention, but not hyperactivity, may be of particular importance in understanding SB and NSSI. Brief screening for impulsivity and inattention in childhood could facilitate detection of children vulnerable to NSSI and SB and indicate valuable information for preventive and intervention strategies.

**Supplementary information:**

The online version contains supplementary material available at 10.1186/s12888-022-04311-5.

## Background

Attention-Deficit/Hyperactivity Disorder (ADHD) is a neurodevelopmental disorder (NDD) characterized by deficient cognitive abilities that appear during childhood [1]. Worldwide, 5.3–7.2% of children are estimated to fulfill criteria for ADHD [2, 3], and these children are at elevated risk of several adverse events as they grow older [4], including self-injury [5, 6]. Self-injury has an average onset between age 13 and 15, and a life-time prevalence of 16.9% in adolescents [7–9]. Self-injurious behaviors include both nonsuicidal self-injury (NSSI) and suicidal behavior (SB) that could be seen as distinct phenomena separated by intention, frequency, and medical lethality [10]. Despite these differences, NSSI and SB can often co-occur and several theories have been put forward on how NSSI and SB might be linked [10]. Children with ADHD seem to be at increased risk of both NSSI and SB [11–13]; in one study including only girls, the odds of NSSI and SB in adolescence/early adulthood were four times greater for girls with ADHD (i.e., the combined subtype) compared to girls without ADHD [13]. Those with the combined subtype of ADHD also seem to be at the greatest risk of the most severe forms and highest frequency of NSSI [14]. In addition, children and adolescents with subsyndromal ADHD could also have an increased risk of NSSI [15]. This is concerning as NSSI and SB are, in turn, associated with a range of adverse outcomes including substance misuse, incidence of other psychopathology, and death by suicide [16–20].

To better understand the association between ADHD and NSSI or SB, the subdimensions of ADHD (impulsivity, hyperactivity, and inattention) might be of relevance. In the current diagnostic criteria of ADHD, hyperactivity and impulsivity are combined [1], but there is evidence that these subdimensions could load onto three separate factors [21, 22]. However, these subdimensions are not exclusive to ADHD; impulsivity and inattention are also symptoms present in other psychiatric disorders, such as depressive and personality disorders [1] and have been suggested as psychiatric transdiagnostic dimensions [23]. Previous cross-sectional studies have indicated that symptoms of hyperactivity-impulsivity, but not inattention, are associated with NSSI among adolescents [24] and SB among children [25]. The same pattern has been found in longitudinal studies of children with ADHD; the combined or primarily hyperactive-impulsive subtype of childhood ADHD have been more strongly associated with NSSI and SB compared to the primarily inattentive subtype [13, 26]. However, in a longitudinal study of girls with and without ADHD in childhood, both symptoms of inattention and hyperactivity-impulsivity were associated with NSSI and SB in adolescence and young adulthood [27]. In summary, it seems as if hyperactivity-impulsivity symptoms are of importance, and the results are mixed regarding the role of inattention, in understanding NSSI and SB. Nevertheless, these prior studies have not distinguished hyperactivity from impulsivity. In fact, there is evidence indicating that impulsivity and hyperactivity load onto two separate factors [21, 22] and show dissimilar associations to other symptoms, such as emotion dysregulation [28] and autistic traits [21]. Hence, there is reason to investigate impulsivity and hyperactivity separately for NSSI and SB. Regarding NSSI and SB, studies on impulsivity alone show positive significant associations [29–31]. Some studies indicate stronger associations between impulsivity and SB relative to NSSI [31], while others do not [30]. Impulsivity is thought to be associated with NSSI and SB through for instance (as summarized in [29]): (1) increasing risk of engagement in easily accessible maladaptive strategies that can rapidly regulate negative emotions (a common function of NSSI [32]), (2) influencing the progression from suicidal intention to SB [33] and, (3) increasing the risk of painful and provocative experiences that may decrease the aversive nature of SB and increase the acquired capability of SB [34]. In contrast to impulsivity, less is known about hyperactivity alone. Altogether, to what extent impulsivity, hyperactivity, and inattention are associated with NSSI and SB prospectively is insufficiently explored, but may be clinically important, given the excess risks of NSSI and SB.

### Objective

The objective of this study was to investigate the associations of childhood impulsivity, hyperactivity, and inattention with NSSI and SB in individuals at risk for NDDs (including ADHD). We hypothesized that impulsivity would be positively associated with both NSSI and SB. Based on the conflicting prior knowledge, potential difference in strength of association between impulsivity and NSSI relative to SB, was investigated with an explorative approach. Furthermore, considering the conflicting and limited prior knowledge regarding inattention and hyperactivity, respectively, these subdimensions were investigated in a more exploratory manner.

## Methods

### Study design

This study has a longitudinal design with a baseline measurement at age 9 or 12 by caregiver/legal guardian via telephone interview, and a follow-up in-person clinician administered assessment at age 15. The study is presented according to the *strengthening the reporting of observational studies in epidemiology checklist (STROBE)* [35].

### Setting

All twins born in 1992 and onwards in Sweden are invited to participate in the *Child and Adolescent Twin Study in Sweden* (CATSS), investigating somatic and mental health in twins [36]. Caregiver(s) (or legal guardian[s], hereafter referred to as caregiver[s]) to twins were asked to participate in a telephone interview when their twins were 12 years old (for twins born between July 1992 and June 1995) or 9 years old (for twins born July 1995 and onwards). The interview was conducted by lay persons recruited from an interview company, with caregivers as informants. Between July 2004 and January 2010 (i.e., the time frame where the sub-sample descibed below was invited) the response rate for age 9 or 12 was 80% [36].

Families from the CATSS cohort with twin pairs at risk of NDDs (see below) were invited to participate in an in-depth clinical assessment at age 15 to investigate outcomes in adolescence. The follow-up study was entitled *Developmental Outcomes for neurodevelopmental problems in a Genetic twin Study in Sweden* (DOGSS; [36]). Those invited to DOGSS were same-sex twin pairs born between 1993 and 1995, where at least one twin screened above cut-off for a NDD (i.e., ADHD, autism spectrum disorder [ASD], learning disorder, tic disorder, developmental coordination disorder) or common comorbidity (i.e., oppositional defiant disorder, conduct disorder, obsessive compulsive disorder, and/or eating disorder) in the Autism–Tics, ADHD, and other Comorbidities inventory (A-TAC) [37, 38] at age 9 or 12. Some screen-negative twin pairs were also invited as matched controls. From the 1995 birthyear CATSS cohort, the eligibility criteria were adjusted to only inviting families where one or both twins were screen-positive for ADHD and/or ASD at age 9 or 12. Families were contacted when the adolescents were 15 years old by mail and a follow-up telephone call, to inquire if the family was interested in participating in DOGSS. In total, 860 participants were invited and 450 (52%) consented and participated in DOGSS. Attrition analyses indicated that the group that declined participation in DOGSS had higher prevalence of boys, higher prevalence of ADHD screen-positive children, higher prevalence of families where at least one caregiver had secondary school education as highest education level, and lower prevalence of families where both caregivers had primary school education or below as highest education level, compared to those participating in DOGSS [39]. The clinical assessments were conducted in Sweden, either in Stockholm, Gothenburg, or Malmö, between 2008 and 2010, by two clinical licensed psychologists blind to prior information about the participant and to the assessments of their co-twin to decrease risk of interviewer bias. A child psychiatrist, blind to the identity of twin-pairs, reviewed each case together with the responsible psychologist, and assessed possible diagnoses based on all available data.

### Participants

As described above, the objective of DOGSS was to, at age 15, follow up twin pairs who were identified to be at risk for NDDs at age 9 or 12. The sample for the current study (N = 404) thus includes twin pairs where one or both twins were screen-positive for NDD or common comorbidity at age 9 or 12.

### Measurements

#### Exposure: ADHD subdimension

The exposures at age 9 or 12 were the degree of the impulsivity, hyperactivity, and inattention, measured with the A-TAC. A-TAC is a fully structured parent-report inventory that was read out to the participants during the interview and has been used and psychometrically evaluated as a telephone interview with lay persons as interviewers [37, 38, 40–43]. A-TAC encompasses theoretically defined problem areas (e.g., flexibility, attention, memory, motor control) within NDDs, rather than diagnostic categories. A-TAC has shown excellent screening properties when compared to diagnoses assigned at clinics and A-TAC is a reliable instrument for identifying and predicting NDDs in childhood [37, 38, 40–43]. The response categories in A-TAC are ‘no’ (0), ‘yes, to some extent’ (0.5), and ‘yes’ (1). In this study, we separated items corresponding to the criteria of ADHD in the International Statistical Classification of Diseases and Health-Related Problems, 10th Revision (ICD-10 [44]); impulsivity (4 items), hyperactivity (5 items), and inattention (9 items), as this was previously shown to be a valid categorization of these items within the A-TAC [21]. See Table S1, for list of items that were included in each subdimension. For the analysis, standardized sum scores of the subdimensions were used.

#### Outcome: NSSI and SB

The outcomes at age 15 were life-time engagement of NSSI and SB, both measured through the semi-structured clinical interview Schedule for Affective Disorders and Schizophrenia for School-Age Children-Present and Lifetime Version (K-SADS-PL) [45], the second version from 2009 [46]. K-SADS-PL has previously shown to perform better in detecting NSSI and SB compared to less structured clinical evaluation [47]. Questions about NSSI and SB are included in the section of depressive symptoms. The clinician asked several questions about NSSI and SB (e.g., engagement in different methods, intention) to both adolescent and caregiver separately. To measure NSSI in this study, a clinician-rating on item 4e “Non-Suicidal Physical Self-Damaging Acts” was used. A frequency of ≥ 1 episode of life-time engagement of NSSI was used to assess presence of NSSI (coded 0 or 1). To measure SB in this study, clinician-ratings on items 4c “Suicidal Acts – Intent” (also including assessment of life-time suicide attempt), and 4d “Suicidal Acts – Medical Lethality” were used. This entails that preparations for suicide attempt, interrupted suicide attempt, or suicide attempt, were operationalized as SB. Life-time engagements in any of these behaviors were used to assess presence of SB (coded 0 or 1). Life-time prevalence for each outcome was chosen to maximize statistical power.

#### Covariates

Age at baseline interview, birthyear, and sex, were adjusted for to account for differences in time points (i.e., age at baseline interview), cohort effects (i.e., birthyear) and sex differences; as associations between ADHD and SB have previously been shown to vary by sex [26]. Based on the overlap of NDD symptoms [21], other NDD symptoms were adjusted for to understand the potential unique association of impulsivity, hyperactivity, and inattention, respectively. To adjust for other NDD symptoms we created a factor score based on sum scores from 9 defined scales (i.e., tics, flexibility, motor control, social interaction, learning, language, memory, perception, and planning and organizing); in a confirmatory one-factor model we generated an individual factor score per participant. A one-factor model was decided on beforehand to capture and account for NDD symptoms and weigh the different scales in a reasonable way, keeping the number of predictors in the model low to retain statistical power. In addition, a single factor has previously shown to account for a large proportion of variation between NDD symptoms [48]. We also tested a two-factor model to adjust for other NDD symptoms and it did not alter the results (results not reported here). This score was then used in the subsequent analyses. Loadings from the factor analysis are found in Table S2.

### Statistical analysis

#### Main analyses

For both outcomes logistic regression was used to calculate odds ratios (OR). To account for the dependence within twin pairs we applied generalized estimating equations (GEE) and calculated cluster robust standard errors [49]. We fitted separate models for NSSI and SB, resulting in the following ten different models: (1) six crude univariable models including impulsivity, hyperactivity, or inattention on NSSI and SB, (2) two multivariable models, including impulsivity, hyperactivity, and inattention simultaneously, on NSSI and SB, and lastly, (3) two confounder-adjusted models including impulsivity, hyperactivity, and inattention simultaneously as well as age at baseline interview, birthyear, sex, and other NDD symptoms, on NSSI and SB. The ADHD subdimensions and other NDD symptoms factor score was added in the model as standardized continuous variables, and sex (female/male), age at interview (9/12), and birthyear (1993/1994/1995) as categorical variables with two or three possible values. Frequency and patterns of missingness were investigated descriptively and graphically. A power analysis was conducted when planning the current study, after the data collection was completed but before the data analysis was conducted and is presented in Appendix S1.

#### Sensitivity analyses

Two sets of sensitivity analyses were conducted. First, the main analyses were repeated with suicide attempt only (i.e., excluding suicidal preparatory behavior and interrupted attempt; see Appendix S2) as the outcome. This sensitivity analysis was conducted to investigate if the patterns of the main results differed between the more inclusive phenomena of suicide behavior and the specific phenomena of suicide attempt. Hence, if the choice of suicidal outcome seemed to impact the results. Second, the main analyses were repeated splitting the data set into two subgroups according to NDD screen-status at age 9 or 12: (1) NDD screen-positive, and (2) NDD screen-negative (either screen-positive for common comorbidity only or screen-negative co-twin). This second set of sensitivity analyses aimed to investigate the generalizability of the results to groups with or without elevated NDD symptoms at baseline.

All analyses were performed with the software R, version 4.0.3 [50]. GEE was applied through the “drgee” package in R [51].

## Results

### Participants

Out of the 404 participants, 13 (3.2%) participants had some missing values on items regarding ADHD, other NDD symptoms, NSSI or SB, of which 3 (0.7%) were missing on the NSSI and/or SB variable. Further description of missingness is found in Figure S1. Based on the low amount of missingness [52], only participants with complete data were included in the analyses. The final sample consisted of 391 participants.

### Descriptive data

A total of 32 (8.2%) adolescents had the outcome of NSSI and 18 (4.6%) adolescents the outcome of SB. A total of 25 (6.4%) adolescents had engaged in NSSI solely, 11 (2.8%) adolescents had engaged in SB solely, and 7 (1.8%) adolescents had engaged in both NSSI and SB. Among those with NSSI, 24 (75.0%) adolescents were girls, and 5 (16%) had ever engaged in NSSI more than four times a year or with significant tissue damage (e.g., left scars or required stitches). Among those with SB, 12 (66.7%) adolescents were girls, and 3 (17%) had, in relation to SB, either needed, or sought medical care, experienced significant bleeding, or taken more than a couple of pills. The characteristics of the total sample and separated by outcome are presented in Table 1. Additional diagnostic information from the clinical assessment at age 15 is presented in Table S3 and indicates that the sample could be seen as semi-clinical (with approximately 50% fulfilling a NDD or psychiatric disorder). The means and standard deviations of the exposures separated by outcome are presented in Table 2.

**Table 1:** Participant characteristics including demographic and clinical information

	*No NSSI or SB (n = 348)*		*NSSI and/or SB (n = 43)*		*Total (n = 391)*	
	*n*	*%*	*n*	*%*	*n*	*%*
**Age at interview**						
9	16	4.6	3	7.0	19	4.9
12	332	95.4	40	93.0	372	95.1
**Birthyear**						
1993	183	52.6	26	60.5	209	53.5
1994	125	35.9	12	27.9	137	35.0
1995	40	11.5	5	11.6	45	11.5
**Sex**						
Female	136	39.1	30	69.8	166	42.5
Male	212	60.9	13	30.2	225	57.5
**Screen status** ^**a**^						
Screen-positive NDD	168	48.3	24	55.8	192	49.1
Screen-positive comorbidity only^b^	42	12.1	6	14.0	48	12.3
Screen-negative co- twin	138	39.7	13	30.2	151	38.6
**Screen-positive disorder** ^**a,c**^						
ADHD	75	21.6	14	32.6	89	22.8
Learning disorder	66	19.0	8	18.6	74	18.9
ODD	35	10.1	10	23.3	45	11.5
DCD	32	9.2	5	11.6	37	9.5
OCD	34	9.8	3	7.0	37	9.5
Tic disorder	31	8.9	4	9.3	35	9.0
Autism spectrum disorder	23	6.7	3	7.0	26	6.7
**Childhood self-harm** ^**a,d**^	9	2.6	4	9.3	13	3.3
	*Mean*	*SD*	*Mean*	*SD*	*Mean*	*SD*
**Other NDD factor** ^**e**^	-0.02	1.01	0.2	0.91	0	1.0

*Note.* ADHD = Attention-Deficit/Hyperactivity Disorder; DCD = Developmental coordination disorder; NSSI = Nonsuicidal self-injury; NDD = Neurodevelopmental disorder; OCD = obsessive-compulsive disorder; ODD = Oppositional defiant disorder; SB = Suicidal behavior; SD = Standard deviation

^a^At age 9 or 12

^b^Oppositional defiant disorder, conduct disorder, obsessive-compulsive disorder, and/or eating disorder

^c^Multiple screen-positive disorders were possible. In addition, 13 participants (3.3%) were screen-positive for eating disorders and 4 (1.0%) participants for conduct disorder

^d^Self-harm measured with a caregiver as respondent in the A-TAC through the item “Has he/she ever deliberately hurt him/herself?”. Both ‘yes, to some extent’ (0.5) and ‘yes’ (1) were considered presence of self-harm. In total, 36 (9.2%) participants had missing values on this item

^e^Standardized factor score generated through factor analysis with the other neurodevelopmental disorder items as described in *Methods* section

**Table 2:** Mean and standard deviation on the exposure variables separated by outcome and for the whole sample

		*No NSSI or SB (n = 348)*		*NSSI (n = 32)*		*SB (n = 18)*		*Total (n = 391)*	
	*Range*	*Mean*	*SD*	*Mean*	*SD*	*Mean*	*SD*	*Mean*	*SD*
Impulsivity	0–4	0.78	1.08	1.23	1.34	1.56	1.43	0.84	1.12
Hyperactivity	0–5	0.71	1.17	0.84	1.39	0.92	1.42	0.73	1.21
Inattention	0–9	2.39	2.60	3.78	2.81	3.47	2.83	2.53	2.64

*Note.* NSSI = Nonsuicidal self-injury; SB = Suicidal behaviors; SD = Standard deviation

### Main analyses

**Fig. 1:**
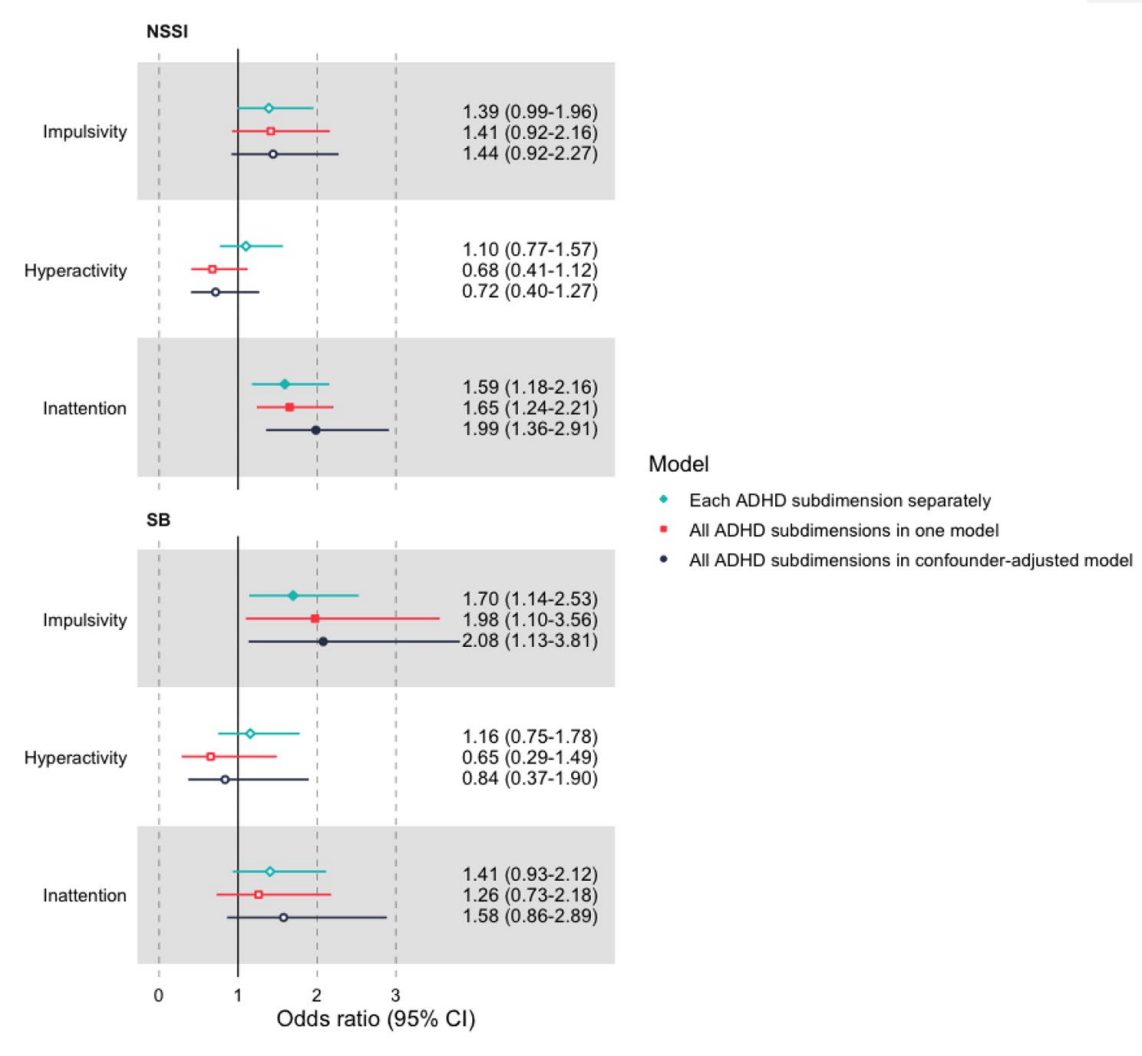
Associations between impulsivity, hyperactivity, or inattention, and nonsuicidal self-injury and suicidal behavior in the three different models. Note. A filled dot indicates a statistically significant effect on the α = 0.05 level. ADHD = Attention-Deficit/Hyperactivity Disorder; NSSI = Nonsuicidal self-injury; SB = Suicidal behaviors

Figure 1 illustrates the OR and 95% confidence intervals obtained from the logistic regression analyses for the univariable, multivariable, and confounder-adjusted models for NSSI and SB. Full results from the confounder-adjusted models are found in Table S4. Childhood impulsivity had a statistically significant positive association with SB in all models, whereas the association was statistically non-significant in all models and somewhat weaker for NSSI. Furthermore, the effect of hyperactivity on both outcomes were not statistically significant and differed in direction of OR between models, with small effects, indicating low impact of hyperactivity on both outcomes. Lastly, childhood inattention had a statistically significant positive association with NSSI in all models, whereas the association was not statistically significant in all models and somewhat weaker for SB.

### Sensitivity analyses

For the outcome of suicide attempts, similar patterns were found compared to the outcome of SB as shown in Table S5. When splitting the data set based on NDD screen status (screen-positive [N = 192] and screen-negative [N = 199]), the results were uncertain with statistically non-significant results in almost all models. Hence, the generalizability of the main results to any of the subgroups specifically could not be confirmed. Results are found in Table S6 and S7.

## Discussion

The objective of this longitudinal study, following children at risk for NDDs into adolescence, was to investigate the associations of childhood impulsivity, hyperactivity, and inattention with NSSI and SB. The results showed that childhood impulsivity was associated with SB, childhood hyperactivity was not associated with either NSSI or SB, and lastly, that childhood inattention was associated with NSSI.

In accordance with our expectations, and results from previous meta-analyses [29–31], impulsivity was associated with SB. Some potential reasons for this finding as well as possible implications are worth highlighting. First, as impulsivity is theorized to impact the progression from suicidal ideation to SB specifically [33], this could imply that it is important to note, and intervene on, high levels of impulsivity in combination with suicidal ideation to decrease risk of SB in childhood or adolescence. Second, it has also been theorized that impulsive individuals could encounter more painful or proactive events relative to non-impulsive individuals; the accumulation of such events could, in turn, decrease the aversive nature of SB and increase the acquired capability of SB [34]. Thus, it could be of importance to pay attention to the occurrence and consequences of painful or proactive events in children with high levels of impulsivity. Moving on to NSSI, the association between impulsivity and NSSI was not confirmed in any of the models. Although the ORs indicated positive associations, in line with our expectations, the confidence intervals contained 1 and it is reasonable to believe that this is a consequence of constrained statistical power. Regarding strength of association, although not formally tested in this study, impulsivity seems more strongly associated to SB compared to NSSI, in line with some previous research [31]. A potential reason for this observation is that childhood impulsivity might impact a faster development from NSSI to SB; of note, 39% of those with SB had a history of NSSI engagement. On the other hand, it could also potentially be that SB is more of an impulsive act within this young population, whereas NSSI to a greater extent is performed more deliberately. Nevertheless, the reason for this trend is a question for future research to address.

Hyperactivity was not associated with either NSSI or SB in this study. These results indicate that impulsivity seems to be the driving factor in the association between impulsivity and hyperactivity combined, and NSSI or SB. This result points at the need to study and consider these ADHD subdimensions separately.

In line with one previous study [27] and contrary to the results from a prior master thesis [24], we found an association between inattention and NSSI. This finding might be understood in the light of emotion regulation theory. Emotion regulation refers to: “the processes by which individuals influence which emotions they have, when they have them, and how they experience and express these emotions” [53, p.275]. In the emotion regulation process, individuals may regulate their emotions by directing their attention away from aversive emotional stimuli or attending alternative stimuli [53]. Individuals with NSSI may have deficits in the executive attention functioning [54], potentially impacting both the process of directing attention away from emotional stimuli as well as attending alternative stimuli. This may lead to continued exposure to aversive emotional stimuli and increased emotional intensity (as described in [54]). In turn, high emotional intensity could lead to greater efforts, adaptive or maladaptive, to regulate emotions [55]. Difficulties to adaptively regulate emotions could increase risk of NSSI [56], functioning as a rapid and maladaptive strategy to regulate emotions in the moment, despite the long-term negative effects [57]. This could potentially – at least in part – explain the observed association between inattention and NSSI. Another potential explanation of the finding is that inattention might indirectly increase risk for NSSI through boredom; inattention can be associated to boredom [58] and NSSI (potentially in a greater degree than SB) could function to escape unwanted internal states (e.g., as boredom) as well as induce a positive state (e.g., to get a “kick”) [32]. Still, given the scarce knowledge, replications are needed. Additional studies could also be helpful to understand the potentially weaker association between inattention and SB relative to NSSI.

In summary, the findings indicate that the associations between the separate ADHD subdimensions and NSSI and SB may vary. The findings inform about opportunities to detect information in childhood associated with self-injurious behaviors with average onset in adolescence. These findings could be clinically meaningful as children at risk of NDDs might be presented to health care where impulsivity and inattention could be screened for and valuable independent of what the child is referred for. To prevent and intervene on NSSI and SB, interventions aiming to increase impulse control and attentional ability could be valuable.

### Limitations

Although this study has several strengths, such as: population-based recruitment, including both boys and girls, and semi-structured clinician-assessed outcome measures with the possibility to distinguish NSSI from SB, there are also some limitations to highlight. First, this study was constrained by statistical power, where some of the associations might not have been confirmed for this reason. Regarding the prevalence of NSSI and SB, the prevalence of SB was similar to previous findings, but the prevalence of NSSI was lower compared to community samples of adolescents up to age 18 [8, 9]. However, for self-injury generally, the prevalence of 11% in this sample was close to the 15% at age 15 that could be expected in community samples [8]. Still, measuring lifetime prevalence at age 15 could underestimate the prevalence of these behaviors, as it is expected that some individuals might have an onset after age 15 [7–9]. However, given that the assessment of NSSI and SB was performed close to the average onset, this could decrease risk of recall bias. Second, regarding the measurement of the outcome, given that lifetime engagement of NSSI and SB were measured, temporal order of the associations (i.e., exposures occurring before outcomes) could not be established. Furthermore, as NSSI and SB were investigated as binary (yes/no) measures, it remains unknown how the results generalize to different severity levels of NSSI and SB. Nevertheless, the descriptive results indicate that most of participants with NSSI and SB belong to a lower severity level (as defined in the K-SADS-PL). In addition, even if assessment of NSSI was done by experienced clinicians, we cannot exclude the possibility that stereotypical behavior has been coded as NSSI. Lastly, the difference in informants between symptoms measured at age 9 or 12, and 15, could potentially have an impact on the rated prevalence of the symptoms. However, adolescent report is particularly important to include for NSSI and SB given that parents might not be aware of such behaviors [59]. Third, individuals were selected into the cohort based on NDD and common comorbidity screen status, potentially impacting the generalizability. Furthermore, the sensitivity analyses could not confirm generalizability of the main results to any of the two subgroups specifically; the point estimates (presented in Table S6 and S7) leads us to hypothesize that both impulsivity and inattention could be of importance in understanding NSSI and SB in both NDD screen-positive and NDD screen-negative. Furthermore, we did not adjust for what disorder the screen-positive twin was positive for, meaning it is unknown if the associations differ by that factor. Fourth, the generalizability could be restricted given that 80% of eligible participants enrolled in CATSS at age 9 or 12, and from the CATSS cohort only 52% of the invited enrolled in DOGSS. Fifth, the generalizability of twins to non-twins might be questioned; however, it seems as if twins do not systematically differ from non-twins on many measures of behaviors and development, including depression and hyperactivity [60, 61]. Sixth, there was no multiple test correction, and the risk of chance findings and false-positive findings might have increased.

### Future directions

Given the novelty of some of the results, and the few studies on the associations between ADHD subdimensions and NSSI and SB, replications are needed. Further research could also extend the knowledge by looking closer at the effects of ADHD subdimensions on NSSI and SB separated by gender, different frequency, and severity levels of NSSI and SB, and the effect on NSSI and SB alone as well as the combination of NSSI and SB.

## Conclusion

In this longitudinal study following children at risk for NDDs over the transition to adolescence, the findings were that: (1) childhood impulsivity was associated with SB, (2) childhood hyperactivity was not associated with either NSSI or SB, and (3) childhood inattention was associated with NSSI. Brief screening of inattention and impulsivity could offer opportunities to detect children vulnerable to NSSI and SB, and these symptoms could inform preventive and intervention strategies.

### Electronic supplementary material

Below is the link to the electronic supplementary material.

Supplementary Material 1. Supportive information

## Data Availability

The datasets used in the current study are available upon reasonable request to the Swedish Twin Registry: https://ki.se/en/research/staff-and-management. Contact the corresponding author in case of assistance or questions.

## Abbreviations

ADHD: Attention-Deficit/Hyperactivity Disorder
ASD: Autism Spectrum Disorder
CATSS: Child and Adolescent Twin Study in Sweden
DOGSS: Developmental Outcomes for neurodevelopmental problems in a Genetic twin Study in Sweden
GEE: Generalized Estimating Equations
K-SADS-PL: Schedule for Affective Disorders and Schizophrenia for School-Age Children-Present and Lifetime Version
NDD: Neurodevelopmental Disorder
NSSI: Nonsuicidal Self-injury
OR: Odds Ratios
SB: Suicidal Behavior
A-TAC: Autism–Tics, ADHD, and other Comorbidities inventory
