# Correction: Associations of impulsivity, hyperactivity, and inattention with nonsuicidal self-injury and suicidal behavior: longitudinal cohort study following children at risk for neurodevelopmental disorders into mid-adolescence

**DOI:** 10.1186/s12888-023-05338-y

**Published:** 2023-11-21

**Authors:** Olivia Ojala, Ralf Kuja-Halkola, Johan Bjureberg, Anna Ohlis, Martin Cederlöf, Eva Norén Selinus, Paul Lichtenstein, Henrik Larsson, Sebastian Lundström, Clara Hellner

**Affiliations:** 1 Centre for Psychiatry Research, Department of Clinical Neuroscience, Karolinska Institutet, & Stockholm Health Care Services, Norra Stationsgatan 69 7th fl., Stockholm, SE-11,364 Sweden; 2 Department of Medical Epidemiology and Biostatistics, Karolinska Institutet, Stockholm, Sweden; 3 Department of Psychology, Stanford University, Stanford, CA USA; 4 Department of Global Public Health, Karolinska Institutet, Stockholm, Sweden; 5 region vastmanland – Uppsala University, Centre for Clinical Research, Vastmanland Hospital, Vasteras, Sweden; 6 the Swedish School of Sport and Health Sciences, Stockholm, Sweden; 7 School of Medical Sciences, Örebro University, Örebro, Sweden; 8 Gillberg Neuropsychiatry Centre, Institute of Neuroscience and Physiology, University of Gothenburg, Gothenburg, Sweden; 9 Centre for Ethics, Law and Mental health (CELAM), Institute of Neuroscience and Physiology, University of Gothenburg, Gothenburg, Sweden

**Correction: BMC Psychiatry (2022) 22:679**

10.1186/s12888-022-04311-5.

Following publication of the original article [1], the authors would like to correct the numbering of in-text citation under the heading Setting.

The incorrect in-text citation is:

All twins born in 1992 and onwards in Sweden are invited to participate in the *Child and Adolescent Twin Study in Sweden* (CATSS), investigating somatic and mental health in twins **[29]**.

The correct in-text citation is:

All twins born in 1992 and onwards in Sweden are invited to participate in the *Child and Adolescent Twin Study in Sweden* (CATSS), investigating somatic and mental health in twins **[36]**.

The original article [1] has been corrected.
