# Supplementary material for: Associations of impulsivity, hyperactivity, and inattention with nonsuicidal self-injury and suicidal behavior: longitudinal cohort study following children at risk for neurodevelopmental disorders into mid-adolescence
Source: BMC Psychiatry. 2022 Nov 3;22:679. doi: 10.1186/s12888-022-04311-5 (PMC9635086; doi:10.1186/s12888-022-04311-5)
Supplement: Supplementary file 1 — Supplementary Material 1. Supportive information [file 12888_2022_4311_MOESM1_ESM.docx]

**Supportive information -**

**Associations of Impulsivity, Hyperactivity, and Inattention with Nonsuicidal Self-injury and Suicidal Behavior: Longitudinal Cohort Study Following Children at risk for Neurodevelopmental Disorders into Mid-Adolescence**

*BMC Psychiatry*

Olivia Ojala, MSc, Ralf Kuja-Halkola, PhD, Johan Bjureberg, PhD, Anna Ohlis, MD, PhD, Martin Cederlöf, PhD, Eva Norén Selinus, MD, PhD, Paul Lichtenstein, PhD, Henrik Larsson, PhD, Sebastian Lundström, PhD, Clara Hellner, MD, PhD

# Correspondence to:

Olivia Ojala, [olivia.ojala@ki.se](mailto:olivia.simonsson@ki.se) Centre for Psychiatry Research, Department of Clinical Neuroscience, Karolinska Institutet, & Stockholm Health Care Services, Region Stockholm, Sweden

**Table S1**

*Items to measure impulsivity, hyperactivity, and inattention from Autism–Tics, ADHD, and other Comorbidities inventory (A-TAC [1, 2]). A-TAC can be downloaded here:* [*https://www.gu.se/en/gnc/gncs-resources/screening-questionnaires/a-tac-screening-questionnaire*](https://www.gu.se/en/gnc/gncs-resources/screening-questionnaires/a-tac-screening-questionnaire)

|  | **Item** |  |
| --- | --- | --- |
| **Impulsivity** |  |  |
|  | 21 | Does s/he talk excessively? |
|  | 22 | Does s/he often blurt out answers before the question has been completed? |
|  | 23 | Does s/he have difficulty awaiting turns? |
|  | 24 | Does s/he often interrupt or intrude on others? |
| **Hyperactivity** |  |  |
|  | 16 | Does s/he have difficulties holding his/her hands and feet still or can s/he not stay seated? |
|  | 17 | Does s/he get up and move about in school or in other situations when s/he is supposed to remain seated? |
|  | 18 | Does s/he often run around and climbs more than his/hers peers? |
|  | 19 | Does s/he have difficulty playing calmly and quietly? |
|  | 20 | Is s/he often ”on the go” or does s/he often act as if ”driven by a motor”? |
| **Inattention** |  |  |
|  | 7 | Does s/he often fail to pay close attention to details or make careless mistakes in schoolwork, or other activities? |
|  | 8 | Does s/he often have difficulty sustaining attention in tasks or play activities? |
|  | 9 | Does s/he often seem not to listen when spoken to directly? |
|  | 10 | Does s/he have difficulty following instructions and to finish tasks? |
|  | 11 | Does s/he often have difficulty organizing tasks and activities? |
|  | 12 | Does s/he often avoid tasks that require sustained mental effort (such as homework)? |
|  | 13 | Does s/he often lose things? |
|  | 14 | Is s/he often easily distracted or disturbed? |
|  | 15 | Is s/he often forgetful in daily activities? |

*Note.* The grouping of questions to subdimension is according to the criteria of ADHD in ICD-10 [3].

**Table S2**

*Factor loadings and uniqueness generated by confirmatory maximum-likelihood factor analysis with one fitted factor and varimax rotation*

| **Module** | **Number of items** | **Factor loadings** | **Uniqueness** |
| --- | --- | --- | --- |
| Tics | 3 | 0.384 | 0.853 |
| Flexibility | 5 | 0.777 | 0.396 |
| Motor control | 1 | 0.269 | 0.928 |
| Social interaction | 6 | 0.774 | 0.401 |
| Learning | 3 | 0.375 | 0.859 |
| Language | 6 | 0.698 | 0.513 |
| Memory | 3 | 0.553 | 0.695 |
| Perception | 5 | 0.646 | 0.583 |
| Planning & organizing | 2 | 0.730 | 0.468 |

*Note.* Module refers to the theoretically defined problem areas from the A-TAC interview. Number of items refers to the number of items in that module which is included in the factor analysis. Uniqueness refers to variance that is unique and not shared with the other variables. A maximum-likelihood factor analysis with one fitted factor and varimax rotation was used.

**Appendix S1**

*Power analysis*

Two sensitivity power analyses (i.e., stating the sample size, desired power, and alpha level) were conducted after data collection was complete and at the start of planning the current study (before data analysis). The sensitivity power analyses were conducted before specifying the specific sample of this current study, and hence conducted on the full DOGSS sample. With a sample size of 450 participants the study had at least 80% power with an alpha level of 0.05 and using a continuous standard normally distributed exposure, to detect an odds ratio (OR) of at least 1.66 per standard deviation in exposure in relation to NSSI, given a 7.0% prevalence of NSSI. The corresponding number for SB was an OR of at least 1.96, given a prevalence of 3.5% of SB. The sensitivity power analyses were conducted in G*Power [4]. In a previous study comparing girls with and without ADHD, ORs between 2.5 and 4.5 were found (e.g., [5]). Although there are differences between this prior study and the present (e.g., population and the measure of exposure), detection of ORs of at least 1.66 and 1.96 was deemed reasonable and the planning of the study continued. No post hoc power analyses were performed as this is not advised [6].

**Appendix S2**

*Info about sensitivity analysis*

As a sensitivity measure, life-time suicide attempts (SA) were assessed as an outcome, through the semi-structured clinical interview Schedule for Affective Disorders and Schizophrenia for School-Age Children-Present and Lifetime Version (K-SADS-PL) [7], the second version from 2009 [8]. The clinician asked several questions (e.g., engagement in different methods, intention) to both adolescent and caregiver separately. To measure SA in this study, clinician-ratings on items 4c “Suicidal Acts – Intent” (also including assessment of life-time suicide attempt), and 4d “Suicidal Acts – Medical Lethality” were used. This entails that SA is operationalized in this study as excluding preparations for or interrupted SA. Presence of life-time suicide attempt was used to assess presence of SA (coded 0 or 1). In total, 14 (3.6%) participants out of the sample of 391 had the outcome of SA.

**Figure S1**

*Information of missing variables.*


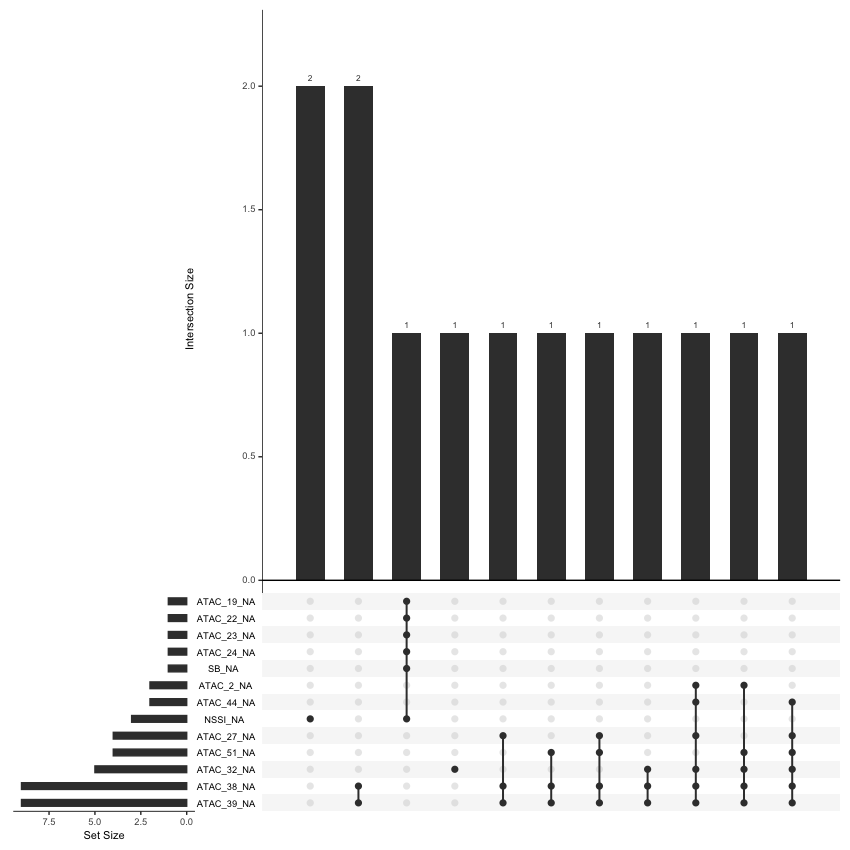


*Note.* Figure illustrates patterns of missingness on the variables with any missing. *Interaction Size* illustrates the number of participants with that missing value or combination of missing values. *Set size* illustrates the total number of missing values on that variable. ATAC_19_NA = Missing on fourth hyperactivity item; ATAC_22_NA = Missing on second impulsivity item; ATAC_23_NA = Missing on third impulsivity item; ATAC_24_NA = Missing on fourth impulsivity item; SB_NA = Missing on the SB item; ATAC_2_NA = Missing on first perception item; ATAC_44_NA = Missing on fifth social interaction item; NSSI_NA = Missing on the NSSI item; ATAC_27_NA = Missing on second learning item; ATAC_51_NA = Missing on the first tics item; ATAC_32_NA = Missing on the second memory item; ATAC_38_NA = Missing on fifth language item; ATAC_39_NA = Missing on the sixth language item

**Table S3**

*Frequency of common psychiatric and neurodevelopmental disorders assessed by clinician and clinical expert at age 15 from the Schedule for Affective Disorders and Schizophrenia for School-Age Children-Present and Lifetime Version (K-SADS-PL) interview^[[1]](#footnote-1)^.*

|  | *No NSSI or SB (n=348)* | | *NSSI and/or SB (n=43)* | | *Total (n=391)* | |
| --- | --- | --- | --- | --- | --- | --- |
|  | *n* | *%* | *n* | *%* | *n* | *%* |
| Disorder |  |  |  |  |  |  |
| ADHD | 69 | 20.1 | 19 | 44.2 | 88 | 22.5 |
| Anxiety^b^ | 58 | 17.2 | 15 | 34.9 | 73 | 18.7 |
| Tic disorder^a^ | 55 | 16.0 | 5 | 11.9 | 60 | 15.3 |
| Depression^c^ | 16 | 4.7 | 16 | 38.1 | 32 | 8.2 |
| ODD/CD | 14 | 4.1 | 13 | 31.7 | 27 | 6.9 |
| PTSD | 20 | 5.9 | 5 | 11.9 | 25 | 6.4 |
| OCD | 13 | 3.8 | 3 | 7.1 | 16 | 4.1 |
| Autism spectrum disorder | 19 | 5.6 | 2 | 4.9 | 21 | 5.4 |
| Eating disorder^d^ | 6 | 1.8 | 4 | 10.0 | 10 | 2.6 |
| Any disorder^e^ | 175 | 51.6 | 39 | 90.7 | 214 | 54.7 |

*Note.* Corresponding to previously or currently fulfilling criteria. ADHD = Attention-Deficit/Hyperactivity Disorder; CD = Conduct disorder; NSSI = Nonsuicidal self-injury; OCD = Obsessive-compulsive disorder; ODD = Oppositional defiant disorder; PTSD = Posttraumatic stress disorder; SB = Suicidal behavior

^a^Tourette’s disorder, persistent (chronic) motor or vocal tic disorder, or provisional tic disorder

^b^ Panic disorder, social phobia, separation anxiety disorder, agoraphobia, specific phobia, or generalized anxiety disorder

^c^Major depressive disorder, dysthymia, or unspecified depressive disorder

^d^Anorexia or bulimia nervosa

^e^Of the disorders listed in the table

**Table S4**

*Associations between impulsivity, hyperactivity, or inattention, and nonsuicidal self-injury and suicidal behavior in the confounder-adjusted model*

|  | ***NSSI*** | | ***SB*** | |
| --- | --- | --- | --- | --- |
|  | *OR* | *95% CI* | *OR* | *95% CI* |
| *IMP* | 1.44 | (0.92-2.27) | 2.08* | (1.13-3.81) |
| *HYP* | 0.72 | (0.40-1.27) | 0.84 | (0.37-1.90) |
| *INATT* | 1.99*** | (1.36-2.91) | 1.58 | (0.86-2.89) |
| *Other NDD symptoms* | 0.83 | (0.58-1.21) | 0.67 | (0.34-1.29) |
| *Sex* | 5.34*** | (2.16-13.21) | 3.15* | (1.02-9.71) |
| *Age at baseline interview* | 0.72 | (0.11-4.64) | 2.21x10^-7^***^a^ | (2.52x10^-8^-1.93x10^-6^) |
| *Birthyear 1994* | 0.62 | (0.22-1.81) | 0.82 | (0.25-2.74) |
| *Birthyear 1995* | 0.76 | (0.23-2.57) | 1.20x10^-7^***^a^ | (2.33x10^-8^-6.18x10^-7^) |

Note. The reference group for sex is male, for age at interview is 9 years old, for birthyear is 1993. Nonsuicidal self-injury; SB = Suicidal behaviors; IMP = Impulsivity; HYP = Hyperactivity; INATT = Inattention

^a^Very little variation among those with the outcome

*P ≤ .05

***P ≤ .001

**Table S5**

*Associations between impulsivity, hyperactivity, or inattention, and suicide attempts in three different models*

|  | **Each ADHD subdimensions separately** | | **All ADHD subdimensions in one model** | | **All ADHD subdimensions in confounder-adjusted model^a^** | |
| --- | --- | --- | --- | --- | --- | --- |
|  | *OR* | *95% CI* | *OR* | *95% CI* | *OR* | *95% CI* |
| IMP | 1.74* | (1.10-2.76) | 2.28* | (1.17-4.41) | 2.48* | (1.22-5.07) |
| HYP | 1.05 | (0.60-1.82) | 0.55 | (0.18-1.66) | 0.78 | (0.23-2.60) |
| INATT | 1.28 | (0.85-1.93) | 1.16 | (0.61-2.19) | 1.51 | (0.72-3.17) |

ADHD = Attention-Deficit/Hyperactivity Disorder; IMP = Impulsivity; HYP = Hyperactivity; INATT = Inattention

^a^Adjusting for sex, birthyear, age at baseline interview, and symptoms of other neurodevelopmental disorder symptoms

*P ≤ .05

**Table S6**

*Associations between impulsivity, hyperactivity, or inattention, and nonsuicidal self-injury and suicidal behavior in the three different models among those screen-positive for neurodevelopmental disorder at age 9 or 12 (N=192)*

|  | **Each ADHD subdimensions separately** | | **All ADHD subdimensions in one model** | | **All ADHD subsimensions in confounder-adjusted model^a^** | |
| --- | --- | --- | --- | --- | --- | --- |
|  | *OR* | *95% CI* | *OR* | *95% CI* | *OR* | *95% CI* |
| **NSSI** |  |  |  |  |  |  |
| IMP | 1.47 | (0.95-2.27) | 1.75 | (0.97-3.15) | 1.81 | (0.99-3.30) |
| HYP | 1.04 | (0.69-1.56) | 0.64 | (0.34-1.21) | 0.70 | (0.33-1.46) |
| INATT | 1.53 | (0.97-2.41) | 1.47 | (0.98-2.22) | 1.76* | (1.13-2.75) |
| **SB** |  |  |  |  |  |  |
| IMP | 1.76* | (1.03-3.03) | 2.16 | (0.92-5.10) | 2.25 | (0.94-5.37) |
| HYP | 1.19 | (0.74-1.90) | 0.74 | (0.28-1.97) | 0.97 | (0.41-2.29) |
| INATT | 1.24 | (0.63-2.42) | 0.99 | (0.43-2.28) | 1.16 | (0.54-2.49) |

ADHD = Attention-Deficit/Hyperactivity Disorder; NSSI = Nonsuicidal self-injury; SB = Suicidal behaviors; IMP = Impulsivity; HYP = Hyperactivity; INATT = Inattention

^a^Adjusting for sex, birthyear, age at baseline interview, and symptoms of other neurodevelopmental disorder symptoms

*P ≤ .05

**Table S7**

*Associations between impulsivity, hyperactivity, or inattention, and nonsuicidal self-injury and suicidal behavior in the three different models among those screen-negative for neurodevelopmental disorder at age 9 or 12 (N=199)*

|  | **Each ADHD subdimensions separately** | | **All ADHD subdimensions in one model** | | **All ADHD subdimensions in confounder-adjusted model^a^** | |
| --- | --- | --- | --- | --- | --- | --- |
|  | *OR* | *95% CI* | *OR* | *95% CI* | *OR* | *95% CI* |
| **NSSI** |  |  |  |  |  |  |
| IMP | 1.01 | (0.52-1.98) | 0.94 | (0.43-2.05) | 0.67 | (0.26-1.70) |
| HYP | 0.76 | (0.27-2.11) | 0.62 | (0.16-2.38) | 0.48 | (0.11-2.15) |
| INATT | 2.24 | (0.96-5.25) | 2.36* | (1.02-5.41) | 2.13 | (0.77-5.88) |
| **SB** |  |  |  |  |  |  |
| IMP | 1.71 | (0.83-3.52) | 1.88 | (0.77-4.56) | 2.06 | (0.79-5.38) |
| HYP | 0.46 | (0.10-2.21) | 0.21 | (0.03-1.64) | 0.26 | (0.05-1.45) |
| INATT | 2.49* | (1.09-5.67) | 2.45 | (0.97-6.18) | 3.44 | (0.82-14.36) |

ADHD = Attention-Deficit/Hyperactivity Disorder; NSSI = Nonsuicidal self-injury; SB = Suicidal behaviors; IMP = Impulsivity; HYP = Hyperactivity; INATT = Inattention

^a^Adjusting for sex, birthyear, age at baseline interview, and symptoms of other neurodevelopmental disorder symptoms

*P ≤ .05

**References**

1. Hansson SL, Svanstrom Rojvall A, Rastam M, Gillberg C, Gillberg C, Anckarsater H (2005) Psychiatric telephone interview with parents for screening of childhood autism - tics, attention-deficit hyperactivity disorder and other comorbidities (A-TAC): Preliminary reliability and validity. The British Journal of Psychiatry 187(3): 262-267. https://doi.org/10.1192/bjp.187.3.262

2. Larson T, Anckarsäter H, Gillberg C, Ståhlberg O, Carlström E, Kadesjö B, . . . Gillberg C (2010) The Autism - Tics, AD/HD and other Comorbidities inventory (A-TAC): further validation of a telephone interview for epidemiological research. BMC psychiatry 10(1): 1-1. https://doi.org/10.1186/1471-244x-10-1

3. World Health Organization (1993) ICD-10 Classification of Mental and Behavioural Disorders: Diagnostic Criteria for Research. Drugs used in Skin Diseases. Albany: World Health Organization.

4. Faul F, Erdfelder E, Buchner A, Lang A-G (2009) Statistical power analyses using GPower 3.1: Tests for correlation and regression analyses. Behavior Research Methods 41(4): 1149-1160. https://doi.org/10.3758/BRM.41.4.1149

5. Hinshaw SP, Owens EB, Zalecki C, Huggins SP, Montenegro-Nevado AJ, Schrodek E, Swanson EN (2012) Prospective Follow-Up of Girls with Attention- Deficit/ Hyperactivity Disorder into Early Adulthood: Continuing Impairment Includes Elevated Risk for Suicide Attempts and Self-Injury. Journal of Consulting and Clinical Psychology 80(6): 1041-1051. https://doi.org/10.1037/a0029451

6. Zhang Y, Hedo R, Rivera A, Rull R, Richardson S, Tu XM (2019) Post hoc power analysis: is it an informative and meaningful analysis? General psychiatry 32(4): e100069-e100069. https://doi.org/10.1136/gpsych-2019-100069

7. Kaufman J, Birmaher B, Brent D, Rao UMA, Flynn C, Moreci P, . . . Ryan N (1997) Schedule for Affective Disorders and Schizophrenia for School-Age Children-Present and Lifetime Version (K-SADS-PL): Initial Reliability and Validity Data. Journal of the American Academy of Child and Adolescent Psychiatry 36(7): 980-988. https://doi.org/10.1097/00004583-199707000-00021

8. Axelson D, Birmaher B, Zelazny J, Kaufman J, Gill M (2009), K-SADS-PL 2009 Working Draft. Western Psychiatric Institute and Clinic: Advanced Center for Intervention and Services Research (ACISR) for Early Onset Mood and Anxiety Disorders.

9. Larson T, Lundström S, Nilsson T, Selinus EN, Råstam M, Lichtenstein P, . . . Kerekes N (2013) Predictive properties of the A-TAC inventory when screening for childhood-onset neurodevelopmental problems in a population-based sample. BMC psychiatry 13(1): 233. https://doi.org/10.1186/1471-244X-13-233

1. Results from other interviews and psychological tests were administered and were available to clinicians when they ascertained disorder. For further information, see Larson T, Lundström S, Nilsson T, Selinus EN, Råstam M, Lichtenstein P, . . . Kerekes N (2013) Predictive properties of the A-TAC inventory when screening for childhood-onset neurodevelopmental problems in a population-based sample. BMC psychiatry 13(1): 233. https://doi.org/10.1186/1471-244X-13-233 [↑](#footnote-ref-1)
